# Cognitive processes are robust to early environmental conditions in two lizard species

**DOI:** 10.1093/beheco/araf048

**Published:** 2025-05-30

**Authors:** Pablo Recio, Dalton C Leibold, Ondi L Crino, Kristoffer H Wild, Christopher R Friesen, Basile Mauclaire, Amelia Y Peardon, Daniel W A Noble

**Affiliations:** Division of Ecology and Evolution, Research School of Biology, The Australian National University, 134 Linnaeus Way, Acton ACT 2601,Australia; Division of Ecology and Evolution, Research School of Biology, The Australian National University, 134 Linnaeus Way, Acton ACT 2601,Australia; Division of Ecology and Evolution, Research School of Biology, The Australian National University, 134 Linnaeus Way, Acton ACT 2601,Australia; Flinders University, College of Science and Engineering, Bedford Park, SA 5042, Australia; Division of Ecology and Evolution, Research School of Biology, The Australian National University, 134 Linnaeus Way, Acton ACT 2601,Australia; The University of Melbourne, School of BioSciences, Parkville VIC 3010, Australia; University of Wollongong, Building 17, Keiraville NSW 2522,Australia; Environmental Futures University of Wollongong, Northfields Ave, Wollongong NSW 2500, Australia; Division of Ecology and Evolution, Research School of Biology, The Australian National University, 134 Linnaeus Way, Acton ACT 2601,Australia; Université de Lille, Av. Paul Langevin Bâtiment, Lille 59000, France; Division of Ecology and Evolution, Research School of Biology, The Australian National University, 134 Linnaeus Way, Acton ACT 2601,Australia; Division of Ecology and Evolution, Research School of Biology, The Australian National University, 134 Linnaeus Way, Acton ACT 2601,Australia

**Keywords:** cognition, corticosterone, incubation temperature, lizard, learning, stress

## Abstract

Animals must acquire new information through learning to adjust their behavior adaptively. However, learning ability can be constrained by conditions experienced during early development, when the brain is especially susceptible to environmental conditions. For example, temperature can result in phenotypically plastic adjustments to growth, metabolism, and learning in ectotherms. In vertebrates, thermal conditions can increase the production of glucocorticoid (GCs) - ‘stress’ hormones. Maternal GCs can be transmitted to offspring during development, potentially impacting their learning abilities. GCs and thermal environments are, therefore, predicted to have interactive effects on the development of learning in ectotherms. Here, we investigated the combined effects of prenatal corticosterone (CORT) - the main GC in reptiles—and incubation temperature on associative learning using two species of lizards, *Lampropholis delicata* and *L. guichenoti*. We manipulated CORT levels and temperature in a 2 × 2 factorial design, and then subjected juveniles to a color-associative learning task. We predicted that elevated CORT and low temperatures would impair associative learning. However, both species showed similar learning rates independently of treatment. Our results suggest that these two species may have evolved mechanisms to maintain learning performance despite prenatal challenges. We also found that color affected decision-making in both species. Overall, we observed a non-learned preference towards blue, underscoring the need to carefully select the color used in cognitive tests involving visual stimuli.

## Teaser

Animals need to learn to adapt their behavior, but early developmental conditions can limit this ability. Factors like temperature and maternal stress hormones (glucocorticoids) may interact to influence learning, particularly in ectotherms. We studied how glucocorticoids and temperature during incubation affected learning in two lizard species. Surprisingly, both species maintained similar learning rates regardless of treatment, suggesting they could overcome early challenges. Additionally, color influenced their decision-making, highlighting the importance of color in cognitive tests

## Introduction

Cognition is a set of processes by which animals gather, preserve, and use information from their environment through perception, learning, memory, and decision-making (Shettleworth 2010). These processes are fundamental to foraging, mate selection, antipredatory strategies, and social behaviors, all of which are crucial for survival and reproduction (Dukas 2004). Learning—acquiring and consolidating new information (Dukas 2004) - is essential for coping with environmental changes by enabling individuals to create new associations between events (Dukas 2004; Leal and Powell 2012; Buchanan et al. 2013). However, the capacity to acquire information varies among individuals, shaped by factors such as age, sex, and the developmental environment (Szuran et al. 1994; Lemaire et al. 2000; Zhu et al. 2004; Amiel and Shine 2012; Amiel et al. 2014; Carazo et al. 2014; Noble et al. 2014). Interindividual differences in learning can lead to unequal responses to environmental conditions, potentially affecting population dynamics (Ward-Fear et al. 2016; Welklin et al. 2024). For example, fast learners may better exploit novel resources or avoid new dangerous stimuli, while those with lower learning capabilities might struggle to adapt to environmental changes (Ward-Fear et al. 2016). Learning rate could affect their survival and reproductive output, ultimately influencing population growth rates and stability (Ward-Fear et al. 2016; Welklin et al. 2024). Therefore, understanding the factors that shape learning is crucial to predict how populations will respond to novel conditions.

Variation in learning is usually considered a product of an individual’s capacity to form associations between stimuli through memory formation (Dukas 2004). However, differences in learning among individuals can also result from innate preferences or perceptual biases that influence how information is acquired and affect the decision-making process (Toure and Reader 2022). The brain’s integration of information and the establishment of new connections is a complex process involving interactions among various nuclei to generate responses (Dukas 2004). Consequently, any impact on specific brain regions or their interactions can directly influence learning through memory formation or alterations in decision-making.

Because the brain is highly susceptible to environmental inputs during early stages of life, developmental conditions are especially relevant in shaping cognitive abilities (Zhu et al. 2004). Impacts on brain development can have long-lasting effects on cognition, potentially influencing an individual’s capacity to learn and adapt to new environments (Lemaire et al. 2000; Zhu et al. 2004; Amiel and Shine 2012; Abayarathna and Webb 2020). For example, the state of the mother can influence offspring phenotype beyond genetic transmission through nest-site selection, provisioning, or the transmission of hormones or nutrients (ie “maternal effects” Moore et al. 2019); and the developing brain is also susceptible to these effects.

Glucocorticoids (GCs) are a class of steroid hormones that are particularly relevant in phenotypic plasticity. In vertebrates, GCs regulate metabolism and maintain homeostasis in response to disturbances (ie ‘the stress response’ Sapolsky et al. 2000; Picard et al. 2014). Under stressful situations, animals react by initiating adaptive physiological and behavioral adjustments mediated by GCs. These GCs can be transmitted directly from parents to their offspring with various effects on offspring phenotype (reviewed by Crino et al. 2023). Elevation in GCs during early stages of development typically results in altered neurogenesis, brain structure, and metabolic activity that, in most cases, are related to decreased learning abilities (Lemaire et al. 2000; Zhu et al. 2004; Du et al. 2009; Eaton et al. 2015; Farrell et al. 2015, 2016). For instance, prenatal stress in rats (*Rattus norvegicus*) suppresses neurogenesis in the dentate gyrus, which is associated with impairments in hippocampal-related spatial tasks (Lemaire et al. 2000). Factors such as sex or the nature of the learning task can also affect the direction and magnitude of the effects of prenatal exposure to elevated GCs (Szuran et al. 1994; Crino et al. 2014; Farrell et al. 2015, 2016; Bebus et al. 2016). Because the effects of prenatal GCs on learning can be context-dependent, it is crucial to conduct studies across diverse taxa and experimental conditions to understand these effects fully.

In addition to the environments experienced by parents, offspring also experience potentially stressful environmental conditions that can interact with or amplify parental effects. In ectotherms, the early thermal environment is a mechanism of phenotypic plasticity, influencing a broad spectrum of traits, including growth, metabolism, or cognition (Amiel and Shine 2012; Amiel et al. 2014; Dayananda and Webb 2017; Noble et al. 2018; Abayarathna and Webb 2020). For instance, Port Jackson sharks (*Heterodontus portusjacksoni*) incubated at warmer temperatures took fewer days to master a numerical learning task than those incubated at cooler (Vila Pouca et al. 2019). In skinks, high incubation temperatures are generally associated with faster learning rates (Amiel and Shine 2012; Amiel et al. 2014; Clark et al. 2014); but velvet geckos (*Amalosia lesueurii*) incubated at temperatures beyond their natural thermal range are worse learners than those incubated at colder temperatures (Dayananda and Webb 2017; Abayarathna and Webb 2020). Changes in neural structure and function likely mediate the effects of incubation temperature in reptiles, as high temperatures increase neural density and metabolic activity in the brain (Coomber et al. 1997; Sakata et al. 2000; Amiel et al. 2017; Beltrán et al. 2021).

GCs can play a pivotal role in determining vertebrate responses to elevated temperatures (Crino et al. 2023), potentially fostering natural interactions between temperature and GCs. Additionally, GCs and temperatures can act upon similar physiological mechanisms (Coomber et al. 1997; Lemaire et al. 2000; Sakata et al. 2000; Zhu et al. 2004; Du et al. 2009; Amiel et al. 2017; Beltrán et al. 2021). Therefore, the effects of GCs and temperature may be interdependent. However, the interactive effects of GCs and temperature on learning abilities remain unexplored, yet understanding of how temperature and GC’s interact is particularly relevant in global climate change (Vinogradov et al. 2024).

Here, we investigated the combined effects of prenatal corticosterone (CORT) - the main GC in reptiles—and incubation temperature on cognition in two species of skinks, the delicate skink (*Lampropholis delicata*) and the common garden skink (*L. guichenoti*). We manipulated CORT levels in the eggs and then incubated them at two different temperatures in a 2X2 factorial design. Post-incubation, juveniles were subjected to a color-associative task to assess their learning abilities. We hypothesized that prenatal CORT levels and thermal environment would impact the learning of an association task. We predicted that individuals exposed to high levels of CORT or low temperatures would learn slower than control individuals or those incubated at higher temperatures (Zhu et al. 2004; Amiel and Shine 2012; Eaton et al. 2015; Amiel et al. 2017). Additionally, we predicted that incubation at high temperatures would mitigate the impact of CORT on learning performance, while cold incubation temperatures were expected to enhance the effects of CORT. The interactive effects of CORT and temperature may occur for two reasons that are not mutually exclusive: first, an increase in temperature leads to an overall rise in neural density (Amiel et al. 2017), thereby counteracting the impact of CORT (see Lemaire et al. 2000; Zhu et al. 2004; Eaton et al. 2015); and second, the elevated metabolic rate associated with higher temperatures could accelerate CORT metabolism, resulting in embryos being exposed to CORT for a shorter time. In contrast, glucocorticoids in endotherms are associated with increased energy demands (eg Rubalcaba and Jimeno 2022), which could lead to higher CORT production in lizards incubated at warmer temperatures. However, previous research on *L. delicata* (Crino et al. 2024) has found that cold-incubated lizards had higher baseline CORT levels, suggesting that cooler incubation temperatures may increase the potential effects of CORT exposure.

## Methods

### Subjects

*Lampropholis delicata* and *L. guichenoti* are small [∼ 35 to 55 mm snout-vent length (SVL)], oviparous, and generalist skinks that usually share the same habitat in suburban areas throughout south-eastern Australia (Chapple et al. 2011). Both species have similar breeding periods, but with some differences in reproductive output: while *L. delicata* lays 1 to 6 eggs in only one clutch per season, *L. guichenoti* clutches are smaller (1 to 5 eggs per clutch), and they usually lay two clutches per season (Joss and Minard 1985; Chapple et al. 2011, 2014). Previous studies exploring behavioral differences between the two species have found *L. delicata* to be more exploratory than *L. guichenoti* (Chapple et al. 2011). However, no differences in learning were observed between the skinks in an associative learning task (Bezzina et al. 2014).

### Husbandry

*Breeding colony*—We tested juveniles from a 2019-established lab breeding colony. A total of 270 and 180 adults of *L. delicata* and *L. guichenoti*, respectively, were housed in plastic containers (41.5 L x 30.5 W x 21 H cm) with six lizards (2 males and 4 females) per enclosure. Enclosures were lined with nonstick matting, shelter, and several small water dishes. Water is given daily, and they were fed approximately 40 mid-size crickets (*Acheta domestica*) per enclosure three days a week. Crickets were dusted with calcium weekly and multivitamins and calcium biweekly. We used a heat chord and a heat lamp following a 12 h light:12 h dark cycle to ensure a temperature gradient. Room temperatures were set to 22 to 24 ºC, and the warm side of enclosures was usually at 32 to 34 ºC.

*Eggs collection and incubation*—Between mid-October 2022 to the end of February 2023, we provided females with a place to lay eggs by means of small boxes (12.5 L x 8.3 W x 5 H cm) containing moist vermiculite. These boxes were placed in one side of the communal enclosures (see above). We checked for eggs in the boxes 3 d a week (Monday, Wednesday, and Friday). After collection, we measured the length and width of eggs with a digital caliper to the nearest 0.1 mm, and mass using a digital scale (OHAUS, Model spx123; ± 0.001 g error). Then, we assigned individual IDs to the clutch and each egg. Eggs were treated with a CORT or control treatment (see CORT and Temperature Manipulation below) and were placed in individual cups (80 mL) with moist vermiculite (12 parts water to 4 parts vermiculite). The cups were covered with cling wrap to retain moisture and left in LABWIT 2X5D-R1160 incubators at two different temperatures (see CORT and Temperature manipulation below) until hatching. Eggs were then checked three times a week for hatchlings.

*Hatchlings* – Immediately after hatching, we measured lizards’ snout-to-vent length (SVL) and tail length (TL) with a ruler to the nearest mm, and mass using a digital scale (OHAUS, Model spx123; ± 0.001 g error). Hatchlings were then housed individually in small enclosures (18.7L x 13.2W x 6.3H cm) provided with nonstick matting, shelter, and a water dish. All care otherwise follows similar protocols to adults (see above).

Two weeks prior to the training phase (see details below), 1.5 to 5 mo old lizards (see details in Statistical Analyses and Supplementary Material) were moved to the experimental arena for acclimatization. The arenas were individual medium-size (41 L x 29.7 W x 22 H cm) plastic containers with a shelter (9 L x 6 W x 1.5 H cm) on one of the sides and a water dish on the other. These arenas were placed in two rooms in 7 different racks that were monitored by 7 different CCTV systems (device model DVR-HP210475), allowing us to record their behavior during the experiment (see details below). Although the conditions in the experimental rooms were identical to the colony room, the number of lizards per species and treatment in each rack was counterbalanced to control for any potential effect of the room or the position of the lizard in the rack. During acclimatization and throughout the experiment, lizards were fed with only one cricket per day dusted with calcium and multivitamins, and water was supplied ad libitum. We provided a temperature gradient by means of a heat cord and heat lamps in a 12 h light: 12 h dark cycle.

### CORT and temperature manipulation

**Fig. 1.**
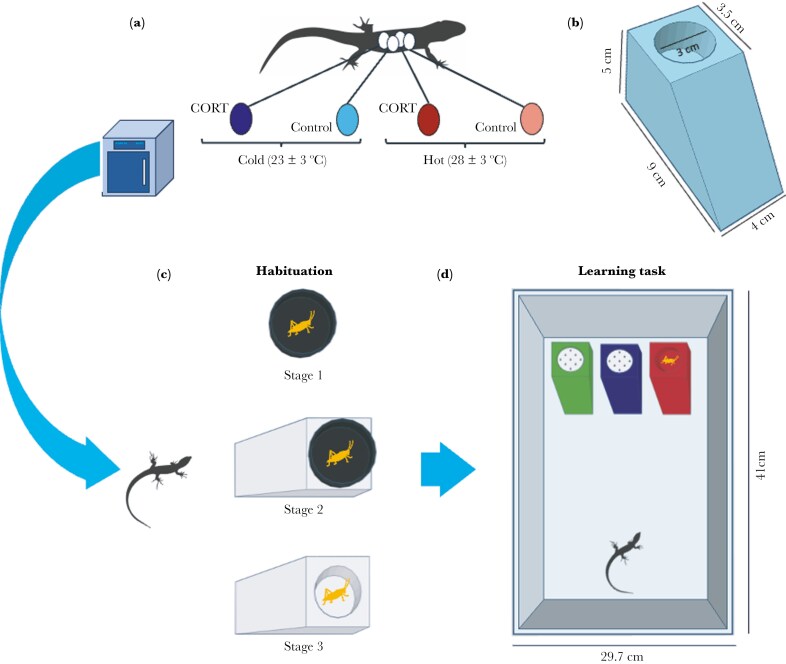
Experimental design of the study. Panel A shows the early environment manipulation. In panel B, the measurements of the 3D-printed ramps that were employed in the habituation and learning tasks. Panel C shows the habituation process in three different stages. In panel D, the associative task is done with the three different 3D-printed ramps. White lids in D show the ramps where the food reward was not attainable.

To empirically test the effect of early environment, we manipulated CORT concentration in eggs and incubated them under one of two temperature regimes (“Cold” - 23 ± 3 ºC or “Hot” - 28 ± 3 ºC) in a 2 × 2 factorial design (Fig. 1A). We topically dosed eggs with a CORT solution (10 pg/mL) or a control treatment (100% ethanol). Corticosterone treatments were made by dissolving crystalline corticosterone (Sigma, Cat. No. C2505) in 100% ethanol. To dose eggs, we applied 5µl of solutions to eggshells using a micropipette. We selected these doses based on previous studies publishing yolk CORT concentrations in other oviparous reptiles (Lovern and Adams 2008; Hanover et al. 2019), while also validating that it fell within the range of CORT concentrations in eggs within our population (Crino et al. 2024). CORT treatment increased mean yolk CORT levels ~3.7x higher than control eggs (Crino et al. 2024). After the treatment, the eggs were incubated in one of the two previously mentioned temperature regimes (“Cold” or “Hot”) until hatching. These temperatures represent the upper and lower limit of the natural incubation temperatures (Qualls and Shine 2000; Cheetham et al. 2011). The number of eggs per clutch assigned to each hormone and temperature treatment was counterbalanced in both species.

### Learning

To estimate learning skills, we tested each lizard’s ability to locate a food reward in a color-associative learning task (Fig. 1C, D). First, we performed a training phase where lizards had to learn to eat from white 3D-printed PLA ramps (9 L x 4 W x 5 H cm) identical in size and shape to the ones used during the associative task (Fig. 1B). We divided the training phase into three stages: the first stage where lizards had to eat a small, frozen cricket (*A. domestica*) from an opaque petri dish (3D x 1.6H cm) placed in the middle of their enclosure (Fig. 1C, Stage 1); the second stage where the petri dish with the cricket was placed on top of the white 3D printed ramps (Fig. 1C, Stage 2); and the third where the cricket was left inside a well (3D x 1.75H cm) on the top of the ramp (Fig. 1C, Stage 3). Every trial began when we left the feeding block (petri dish, ramp, or both) inside the enclosure and finished 1 h later when we took it away. At the end of each trial, we recorded whether the cricket had been consumed. A trial was considered successful if the lizard could locate and consume the reward, while completion of each stage required the lizards to eat the crickets in at least 5 out of 6 trials to ensure lizards were feeding consistently. This phase lasted 38 d until all the lizards learned to eat from the ramp; only in one case did we decide not to use the lizard because its behavior inconsistent throughout the course of the training phase.

During the associative learning phase, we trained lizards to associate color with a food reward (Fig. 1D). This test was like the third stage of the training phase, but here, lizards were presented with three 3D-printed ramps that differed in color. Ramps were green, red, and blue, as previous studies demonstrate that squamates can discriminate between these colors (Baden and Osorio 2019). The food reward (small, frozen, *A. domestica* crickets) was placed inside the wells of the three ramps, covering two of the crickets with 3D-printed lids (3D x 0.5H cm) so prey was only accessible in “the correct” ramp. This way, we controlled for prey chemical cues, as the lids had a series of small holes on the top to allow the release of those chemicals. To control for potential color preference that could bias our results, we split the subjects into two groups counterbalanced by treatment and species: in one group the correct choice (ie the ramp with the non-covered frozen cricket) was blue, while the other group was assigned the red ramp as correct. In all trials, the position of the ramps was changed randomly to ensure subjects were using color rather than spatial cues for the association. Lizards were tested in this task once a day for 35 d.

The experiment occurred between the 6^th^ of March to the 17^th^ of May 2023, and tests were performed between 1100 to 1200 when the lizards were active. Trials in the learning phase were recorded with CCTV systems, always using the same camera per individual. All the videos were analyzed by the same observer (PR) who was blind to the treatment of the lizards. We recorded whether the animal chose the correct ramp first or not. We considered that a choice was made if the head of the lizard was inside the well of one of the ramps. We considered a trial to have failed if there was no choice after 1 h of recording. These trials were scored as NA. We excluded from our analyses those individuals that performed inconsistently, as defined as not choosing in less than 20 out of 35 trials (~57%). We considered each lizard’s first trial to be the first one where a choice was made.

### Statistical analyses

We performed analyses for each species separately. Preliminary analyses showed a significant effect of the color. As such, we decided to split the data by color (blue or red). Therefore, we ran a total of four different Bayesian multilevel models using the *brm* function from the brms package (Bürkner 2017) in R (version 2.8.2) (R Core Team 2021). We ran four parallel MCMC chains of 3000 iterations for each model, with a warmup period of 1000 iterations. We checked that all MCMC chains converged (R_hat_ < 1.2) and mixed effectively to ensure we had > 1000 effective samples from the posterior distribution.

We modeled correct choice [correct (1) or not (0)] as the response variable, and trial, incubation temperature (Cold versus Hot), hormone (CORT versus Control), and the three-way interaction as fixed factors. The error structure was modeled using a Bernoulli distribution with a logit link function [family = Bernoulli(link = ‘logit’)]. We included each lizard’s random intercept and slope (trial) in our models. We also incorporated the clutch identity as a random factor. *L. delicata* lays one clutch per breeding season while *L. guichenoti* lays up to two (Joss and Minard 1985; Chapple et al. 2011, 2014). Since egg collection was done during half of the breeding season, each clutch likely came from a unique mother, so clutch identity captures potential maternal effects. In addition, previous studies indicate that clutches are typically fertilized by a single male, although sperm storage can occur (Kar et al. 2024). Considering our partial split-clutch design and the expectation that maternal effects are likely more pronounced than paternal effects in these species, incorporating clutch as a random factor should effectively account for parental condition.

Learning in lizards can be age-dependent (Noble et al. 2014), and given that incubation temperature will affect hatching time (Zhang et al. 2023), we explored the effects of age on learning by including it as a predictor. Lizard ages at the beginning of the experiment ranged from 41 to 148 d old in *L. delicata* and 48 to 132 in *L. guichenoti*. However, when this variable was included in the models, we did not find any significant effect of age (see *Supplementary Material*). As such, we present models without age as a fixed effect.

Learning rates were the estimated slopes of “trial” and its interaction with hormone and temperature treatments. We used the posterior distributions of parameters to test for differences in learning rates between treatments and species. Slopes directly measure the change in the probability of choosing the correct dish across trials. Learning rates greater than zero, indicating that lizards were improving over trails, were considered evidence of learning, while those less or equal to zero were not.

Decision-making, in contrast, is considered the average probability of choosing correctly in a given trial. We estimated the predicted probability of choosing the correct ramp in the first trial using the intercepts from our posterior distributions following the formula:


$$\begin{array}{l} Probability_{\left(Trial\right)}=\frac{e^{\left(Intercept\;\;\;+\;\;\;Slope\;\;\;\times \;\;\;Trial\right)}}{1+e^{\left(Intercept\;\;\;+\;\;\;Slope\;\;\;\times \;\;\;Trial\right)}} \end{array}$$

In our case, we were interested in estimating the probability of correct choice at the early stages of learning [P_(1)_] because perception can play a role in the learning process (Toure and Reader 2022). It also allowed us to test if lizards were biased towards the assigned color.

We calculated the p_mcmc_ to test the hypothesis that learning rates and contrasts were different from zero, and that the probability of choosing correctly in the first trial was higher than expected by chance (0.33). We considered statistical significance as p_mcmc_ < 0.05.

## Results

**Fig. 2.**
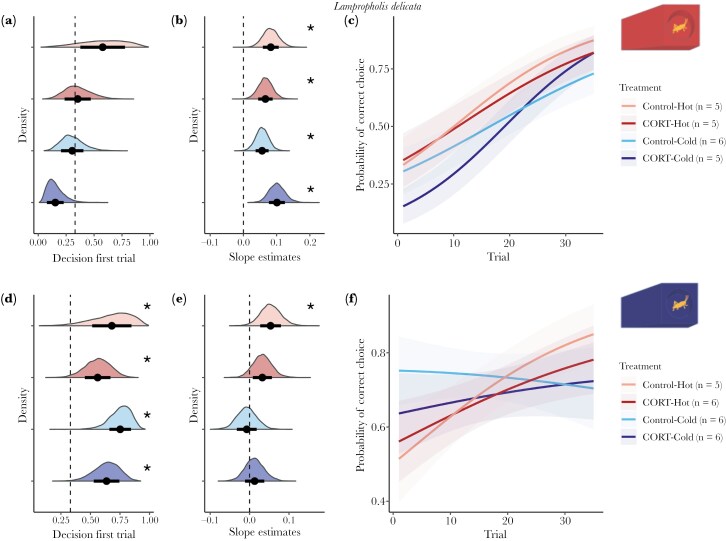
—Results for *Lampropholis delicata* for both color groups red (A, B, C) and blue (D, E, F). Panels A and D show the predicted probability of choosing the correct ramp in the first trial (Decision first trial). In panels B and E, the distribution of the estimates of slopes per each treatment. In all A, B, D, and E the x-axis represents the slope estimate, and in the y-axis are the density of the estimates. The different colors indicate the different treatments. Points and bars represent the mean and standard deviation of the mean of the estimates, respectively. Dashed lines indicate value 0.33 (the probability of choosing correctly by chance) in panels A, D, and 0 in panels B, E. Asterisks indicate significant results (p_mcmc_ < 0.05). Panels C and F show the predicted probability of choosing the correct ramp first over trials. The lines represent the mean predicted probability of choosing the correct ramp for each trial, and the shaded areas indicate the standard deviation of the mean; both were obtained using the slope and intercept estimates from the posterior distributions. The different colors indicate the different treatments.

**Fig. 3.**
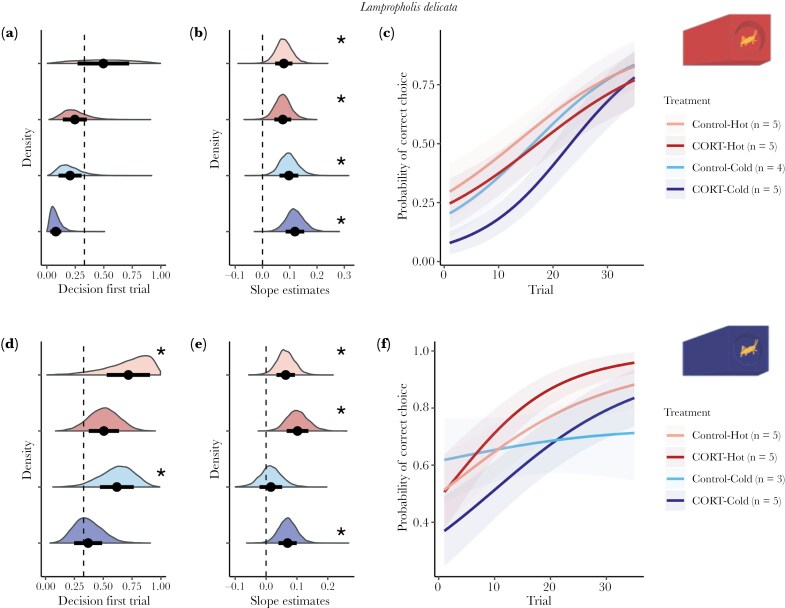
—Results for *Lampropholis guichenoti* for both color groups red (A, B, C) and blue (D, E, F). Panels A and D show the predicted probability of choosing the correct ramp in the first trial (Decision first trial). In panels B and E, the distribution of the estimates of slopes per each treatment. In all A, B, D, and E the x-axis represents the slope estimate, and in the y-axis are the density of the estimates. The different colors indicate the different treatments. Points and bars represent the mean and standard deviation of the mean of the estimates, respectively. Dashed lines indicate value 0.33 (the probability of choosing correctly by chance) in panels A, D, and 0 in panels B, E. Asterisks indicate significant results (p_mcmc_ < 0.05). Panels C and F show the predicted probability of choosing the correct ramp first over trials. The lines represent the mean predicted probability of choosing the correct ramp for each trial, and the shaded areas indicate the standard deviation of the mean; both were obtained using the slope and intercept estimates from the posterior distributions. The different colors indicate the different treatments.

We started with 96 lizards, 48 per species and 12 per treatment per species. However, due to mortality (n = 11), failing to pass the training stage (n = 1), or lack of motivation during the learning tasks (n = 3), we had a final sample size of 81 lizards. Final sample sizes per treatment and species are listed in Fig. 2 and Fig. 3 (figures for both species with the raw data are included in the *Supplementary Material*). Mean slopes (denoted as β throughout) per treatment for both species are provided in Table 1 in the *Supplementary Material*.

### Early developmental environments do not influence visual-sensory systems to impact decision-making

#### Lampropholis delicata.

individuals given the blue ramp as the correct choice had an 89.5% increase in the probability of choosing the right ramp on trial 1 compared to lizards assigned to the red ramps (mean P_Blue_ = 0.657, 95% CI P_Blue_ = [0.361, 0.896]; mean P_Red_ = 0.347, 95% CI P_Red_ = [0.069, 0.835]), suggesting a bias towards blue. A blue bias was also supported by initial choices differing significantly from chance, and this was consistent across all treatments (ie P > 0.33; Table 2 in *Supplementary Material*). However, this difference in the probability of correctly choosing between red and blue ramps was not significant, likely due to small sample sizes (Contrast between probabilities: P_Blue_—P_Red_ = 0.310, p_mcmc_ = 0.23, see Fig. 2).

Decision-making by *L. delicata* was not impacted by the CORT (P_Control_—P_CORT_ = 0.188, p_mcmc_ = 0.20), incubation temperature (P_Hot_—P_Cold_ = 0.237, p_mcmc_ = 0.14) or their interaction ([(P_Control-Hot_—P_CORT-Hot_) - (P_Control-Cold_—P_CORT-Cold_)] = 0.072, p_mcmc_ = 0.20) when red ramps were the correct choice (see Fig. 2A). When blue ramps were the correct choice, there was no significant effect of hormone (P_Control_—P_CORT_ = 0.118, p_mcmc_ = 0.39), temperature (P_Hot_—P_Cold_ = −0.073, p_mcmc_ = 0.65) or their interaction ([(P_Control-Hot_—P_CORT-Hot_) - (P_Control-Cold_—P_CORT-Cold_)] = 0.005, p_mcmc_ = 0.75) either (see Fig. 2D).

#### Lampropholis guichenoti.

when the correct ramps were blue, *L. guichenoti* had a 115.7% increase in the probability of choosing the right ramp on trial 1 compared to red ramps (mean P_Blue_ = 0.553, 95% CI P_Blue_ = [0.201, 0.927]; mean P_Red_ = 0.256, 95% CI P_Red_ = [0.028, 0.806]). However, the difference was not significant (Contrast between probabilities: P_Blue_—P_Red_ = 0.297, p_mcmc_ = 0.16, see Fig. 3).

Decision-making by *L. guichenoti* when the red ramps are the correct choice, was not affected by the hormone treatment (P_Control_—P_CORT_ = 0.188, p_mcmc_ = 0.20), temperature treatment (P_Hot_—P_Cold_ = 0.230, p_mcmc_ = 0.10) or their interaction ([(P_Control-Hot_—P_CORT-Hot_) - (P_Control-Cold_—P_CORT-Cold_)] = 0.125, p_mcmc_ = 0.25; see Fig. 3A). When blue ramps were the correct choice, neither CORT (β_Control_ - β_CORT_ = 0.231, p_mcmc_ = 0.21) incubation temperature (β_Hot_ - β_Cold_ = 0.118, p_mcmc_ = 0.43;), or their interaction ([(P_Control-Hot_—P_CORT-Hot_) - (P_Control-Cold_—P_CORT-Cold_)] = −0.037) have significant effects (Fig. 3D).

### Early developmental environments do not impact the rates of learning across species

#### Lampropholis delicata.

Learning rates for those *L. delicata* assigned to red ramps did not show any significant effects of CORT (β_Control_ - β_CORT_ = −0.014, p_mcmc_ = 0.76), incubation temperature (β_Hot_ - β_Cold_ = −0.004, p_mcmc_ = 0.94), or their interaction ([(β_Control-Hot_ - β_CORT-Hot_) - (β_Control-Cold_ - β_CORT-Cold_)] = 0.061, p_mcmc_ = 0.17) (Fig. 2B, C). Similarly, those assigned to blue were also not affected by CORT (β_Control_ - β_CORT_ = 0.000, p_mcmc_ = 0.98), temperature (β_Hot_ - β_Cold_ = 0.040, p_mcmc_ = 0.32), or their interaction ([(β_Control-Hot_ - β_CORT-Hot_) - (β_Control-Cold_ - β_CORT-Cold_)] = 0.041, p_mcmc_ = 0.41) (Fig. 2E, F).

#### Lampropholis guichenoti.

Learning rates in *L. guichenoti* were not influenced by hormone treatment (β_Control_ - β_CORT_ = −0.009, p_mcmc_ = 0.84), temperature treatment (β_Hot_ - β_Cold_ = -0.032, p_mcmc_ = 0.49), or their interaction ([(β_Control-Hot_ - β_CORT-Hot_) - (β_Control-Cold_ - β_CORT-Cold_)] = 0.026, p_mcmc_ = 0.66) when red ramps were the correct choice (Fig. 3B, C). Similarly, CORT (β_Control_ - β_CORT_ = −0.046, p_mcmc_ = 0.31), incubation temperature (β_Hot_ - β_Cold_ = 0.040, p_mcmc_ = 0.38), or their interaction ([(β_Control-Hot_ - β_CORT-Hot_) - (β_Control-Cold_ - β_CORT-Cold_)] = 0.016, p_mcmc_ = 0.79) were not significant for *L. guichenoti* assigned to blue ramps (see Fig. 3E, F).

### Contrasting impacts of early developmental environments on decision-making and learning in *Lampropholis delicata* and *Lampropholis guichenoti*

Overall, decision-making did not differ between species when the red ramps (mean P_*L. delicata*_ = 0.347, 95% CI P_*L. delicata*_ = [0.069, 0.835]; mean P_*L. guichenoti*_ = 0.256, 95% CI P_*L. guichenoti*_ = [0.028, 0.806]; Contrast between probabilities: P_*L. delicata*_—P_*L. guichenoti*_ = 0.091, p_mcmc_ = 0.52) or the blue ramps were the correct choice (mean P_*L. delicata*_ = 0.657, 95% CI P _*L. delicata*_ = [0.361, 0.896]; mean P_*L. guichenoti*_ = 0.553, 95% CI P_*L. guichenoti*_ = [0.201, 0.927]; P_*L. delicata*_—P_*L. guichenoti*_ = 0.105, p_mcmc_ = 0.61).

We also found no significant differences in learning rates between species when the red ramp (mean β_*L. delicata*_ = 0.077, 95% CI β_*L. delicata*_ = [0.026, 0.135]; mean β_*L. guichenoti*_ = 0.092, 95% CI β_*L. guichenoti*_ = [0.023, 0.171]; Contrast between slopes: β_*L. delicata*_ - β_*L. guichenoti*_ = −0.016, p_mcmc_ = 0.72) or blue ramp was the correct choice (mean β_*L. delicata*_ = 0.023, 95% CI β_*L. delicata*_ = [−0.041, 0.090]; mean β_*L. guichenoti*_ = 0.063, 95% CI β_*L. guichenoti*_ = [−0.030, 0.149]; Contrast between slopes: β_*L. delicata*_ - β_*L. guichenoti*_ = −0.040, p_mcmc_ = 0.40).

## Discussion

We predicted that elevated CORT levels in eggs or incubating them at colder temperatures would decrease learning rates, and that warmer incubation temperatures would suppress the negative effects of CORT on learning. In contrast to our predictions, both species learned to associate a color with a food reward at the same rate regardless of the experimental treatment. These findings suggest that both species may have developed mechanisms to buffer against early environmental stressors and maintain learning performance on color-associative tasks despite prenatal challenges. While we did not find any effect of treatment on decision-making, there was a clear bias towards the color blue irrespective of the treatment, though this pattern differed between the species. We discuss the implications of these findings below.

### Learning is not impacted by prenatal corticosterone exposure

Prenatal CORT was predicted to have a negative effect on learning (Lemaire et al. 2000; Zhu et al. 2004; Eaton et al. 2015; Farrell et al. 2015; Bebus et al. 2016). However, we found no significant difference in learning rates between hormone treatments in either species. Our results are consistent with other experiments that showed no influence of prenatal GCs on learning abilities (Szuran et al. 1994; Bebus et al. 2016; Farrell et al. 2016) and suggest that *L. delicata* and *L. guichenoti* may have developed strategies to buffer the impacts of prenatal stress.

Alternatively, our observations could be obscured by other variables that are known to influence the extent and direction of the effects of GCs (Szuran et al. 1994; Crino et al. 2014; Farrell et al. 2015, 2016; Bebus et al. 2016). GCs are known to have hormetic effects (Du et al. 2009). Generally, low concentrations or short exposure to GCs have been shown to improve the rate at which animals learn while high concentrations or exposure to GCs during long periods have the opposite effects (Du et al. 2009; McEwen 2012). Thus, high elevations in CORT could affect learning through permanent changes in brain function (programmatic effects), while lower doses can result in lifelong elevation of baseline CORT, affecting learning through activational effects. Our findings indicate that associative learning in *L. delicata* and *L. guichenoti* is robust to the programmatic effects of elevated CORT exposure. Similarly, exposing embryos at different stages of development could impact the effects of CORT. However, we cannot exclude the possibility that concentrations different from the one used here or exposure at different developmental times does affect learning.

Conversely, the absence of significant effects of prenatal CORT on learning could be due to the cognitive task employed. Some studies show that the impact of GCs can vary between brain regions (Lemaire et al. 2000). GC actions involve genomic and nongenomic mechanisms that implicate different types of receptors that can be distributed differentially in the various regions of the brain (McEwen 2012), and may be related to the learning performance in distinct tasks. For instance, in European starlings (*Sturnus vulgaris*), males stressed during early stages of development performed worse in a visual associative learning task, but show no differences with control birds with acoustic stimuli (Farrell et al. 2015). Similarly, scrub-jays (*Aphelocoma coerulescens*) with lower CORT levels as nestlings performed better on an associative learning test as adults but not on a reversal-learning task (Bebus et al. 2016). Our results suggest that prenatal CORT does not affect the brain regions involved in coding associative learning in *L. delicata* and *L. guichenoti*, but we cannot rule out the possibility that other cognitive domains, such as spatial learning, might be affected. Future studies should examine the differential effects of early-life stress on different cognitive domains.

### Incubation temperature does not affect learning

We predicted hot-incubated lizards would perform better in the associative learning task, since most studies in other species demonstrate enhanced learning abilities when eggs are incubated at higher temperatures (Amiel and Shine 2012; Amiel et al. 2014; Clark et al. 2014). These studies employed incubation temperatures within natural nesting thermal limits. In contrast, in those studies where cold-incubated lizards outperformed hot incubated ones, the incubation temperatures employed in the hot condition were far above the natural thermal range of the species (Dayananda and Webb 2017; Abayarathna and Webb 2020). As such, it is unclear how such these extreme conditions relate to wild environments. In *L. delicata* and *L. guichenoti*, associative learning appears robust to incubation temperature because of the lack of detectable effect temperature had on learning. Our results, therefore, do not align with previous studies. However, the effect of prenatal temperature on cognition, and brain physiology and structure has been investigated only in a small number of species (see Coomber et al. 1997; Sakata et al. 2000; Amiel and Shine 2012; Amiel et al. 2014, 2017; Clark et al. 2014; Dayananda and Webb 2017; Abayarathna and Webb 2020), limiting our understanding on how early thermal environment can affect cognitive abilities. Our results suggest that the impact of incubation temperature on learning may not be as widespread across reptiles as we think.

### Visual-sensory bias is not dependent on early environment

We found strong evidence for a visual/sensory bias towards choosing the blue feeding ramp during the initial stages of decision-making. While this effect appeared stronger in *L. delicata* compared to *L. guichenoti*, the bias did not result from experiencing different early environmental conditions. What created an initial bias towards blue? One possibility might be that the bias towards blue ramps was a byproduct of the training phase where the blue ramp was more similar to the white training ramp than the green or red ramps. However, this hypothesis is unlikely given that the light spectrum and the perceived chromatic differences between the ramps used in the associative task and the ones used during training phase meant that the white training phase ramp was more similar to the green color rather than blue (see *Supplementary Material*). It seems more likely that lizards innately prefer the blue ramps. Some animals bias their attention towards colors they are familiar with (Putman et al. 2017—the ‘species confidence hypothesis’). For instance, dark and light blue T-shirts were associated with lower flight initiation distances and higher capture rates in Western fence lizards where blue is used in intraspecific communication (Putman et al. 2017). Nevertheless, to the best of our knowledge, there has been no reported color bias in *L. delicata* or *L. guichenoti* before; and blue coloration is not considered to be involved in intraspecific communication in these species (Torr and Shine 1996; Chapple et al. 2014). Regardless, our results demonstrate that, at least in *L. delicata*, there is a bias toward blue colors, highlighting the need to consider the colors used in associative learning paradigms carefully.

### Learning rates between species did not vary

We did not see any difference in learning rates when comparing both species. Since both species occupy similar habitats and have similar ecology (Chapple et al. 2011, 2013, 2014), it may not be surprising that cognitive abilities are similar between both species. Nonetheless, previous studies have shown that *L. guichenoti* is less prone to explore novel environments than *L. delicata*, which may be related to the success of *L. delicata* as an invasive species compared to *L. guichenoti* (Chapple et al. 2011). Our results show that the ability of *L. delicata* to colonize new areas seems not to be related to learning abilities. Our findings support previous studies that found similar learning abilities in *L. delicata* and *L. guichenoti* (Bezzina et al. 2014). In the experiment conducted by Bezzina et al. (2014), both species failed to complete the associative learning task under the authors’ criterion, while in our experiment, both species completed the task, exhibiting similar learning rates. Complexity, experimental design, or the criterion employed to define learning could be the major cause of the discrepancies between ours and Bezzina et al. (2014). Developing common strategies and approaches to assess animal learning could help understand how learning abilities are shaped in different taxa or environments.

## Conclusion

Our results revealed that associative learning abilities and decision-making in *L. delicata* and *L. guichenoti* are resilient to prenatal CORT and temperature. This outcome contrasts with our initial predictions, indicating that the learning skills of these lizards may be more robust than anticipated under varying early life conditions. We also found significant effects of the color employed in the task on learning rates in most *L. delicata* and some *L. guichenoti*. These results seem to be a consequence of an innate color bias and highlight the importance of carefully selecting the color employed when testing cognition using visual stimuli.

Future research should continue exploring the potential effects of prenatal corticosterone (CORT) and temperature on other cognitive functions. Furthermore, it is crucial to explore how these treatments influence brain function at a neurological level. Investigating these aspects will help us understand these species’ cognitive and physiological mechanisms underpinning adaptability and offer insights into how early developmental factors shape long-term cognitive outcomes.

## Supplementary Material

araf048_suppl_Supplementary_Material

## Data accessibility

Analyses reported in this article can be reproduced using the data provided by Recio et al. (2025). All data, data description, and R code are available in public repository: https://github.com/Pablo-Recio/CORT_Temp_learning.
