# Supplementary material for: Cognitive processes are robust to early environmental conditions in two lizard species
Source: Behav Ecol. 2025 May 30;36(4):araf048. doi: 10.1093/beheco/araf048 (PMC12202996; doi:10.1093/beheco/araf048)
Supplement: araf048_suppl_Supplementary_Material [file araf048_suppl_supplementary_material.docx]

# Suplementary Material

#### Estimates of learning rates for all the different treatments per each task, species, and group.

Table 1. Estimates of learning rates for all the different treatments per each task, species, and group. Mean shows the arithmetic means of the estimates obtained from the posteriors of the model, and 95% CI indicates the 95% confidence interval of the mean. All p_mcmc_ tested the hypothesis that the mean equals zero. In bold, those values that are significant (p_mcmc_ <0.05).

|  | | Red | | | Blue | | |
| --- | --- | --- | --- | --- | --- | --- | --- |
| Species | Treatment | Mean | 95% CI | pmcmc | Mean | 95% CI | pmcmc |
| *L. delicata* | CORT-Cold | **0.101** | **[0.055 , 0.152]** | **< 0.001** | 0.013 | [-0.035 , 0.064] | 0.60 |
|  | Control-Cold | **0.056** | **[0.018 , 0.096]** | **< 0.05** | -0.007 | [-0.056 , 0.042] | 0.77 |
|  | CORT-Hot | **0.066** | **[0.023 , 0.110]** | **< 0.05** | 0.032 | [-0.013 , 0.083] | 0.17 |
|  | Control-Hot | **0.082** | **[0.037 , 0.131]** | **< 0.001** | **0.053** | **[0.002 , 0.107]** | **< 0.05** |
| *L. guichenoti* | CORT-Cold | **0.119** | **[0.054 , 0.190]** | **< 0.05** | **0.070** | **[0.012 , 0.129]** | **< 0.05** |
|  | Control-Cold | **0.097** | **[0.032 , 0.171]** | **< 0.05** | 0.015 | [-0.058 , 0.091] | 0.65 |
|  | CORT-Hot | **0.074** | **[0.014 , 0.139]** | **< 0.05** | **0.102** | **[0.036 , 0.177]** | **< 0.05** |
|  | Control-Hot | **0.078** | **[0.016 , 0.145]** | **< 0.05** | **0.064** | **[0.004 , 0.125]** | **< 0.05** |

#### Color preference

To test if lizards were biased towards the assigned color as our preliminary analyses suggested, we employed the values from our posterior distributions. We estimated the predicted probability of choosing the correct ramp first in the first trial (see Statistical analyses) and tested the hypothesis that this estimated probability was higher than expected by chance (i.e. 0.33) using p_mcmc_. If the estimated probability is above 0.33 we consider it as an indication that there was a preference towards that color that could be affecting learning rates.

Table 2. Probability of choosing the correct ramp in the first trial when the correct ramp was blue (Prob Blue) or red (Prob Red) for each species and each treatment. p_mcmc_ tested the hypothesis that the probability is >0.33. In bold, those values that are significant (p_mcmc_ <0.05)

| Species | Treatment | Prob Red | pmcmc Red | Prob Blue | pmcmc Blue |
| --- | --- | --- | --- | --- | --- |
| *L. delicata* | CORT-Cold | 0.153 | 0.97 | **0.636** | **< 0.05** |
|  | Control-Cold | 0.304 | 0.63 | **0.752** | **< 0.001** |
|  | CORT-Hot | 0.353 | 0.45 | **0.561** | **< 0.05** |
|  | Control-Hot | 0.577 | 0.13 | **0.681** | **< 0.05** |
| *L. guichenoti* | CORT-Cold | 0.079 | 1.00 | 0.369 | 0.41 |
|  | Control-Cold | 0.204 | 0.89 | **0.618** | **< 0.05** |
|  | CORT-Hot | 0.246 | 0.81 | 0.506 | 0.09 |
|  | Control-Hot | 0.496 | 0.26 | **0.718** | **< 0.05** |

On average, we found that, for both species, the proportion of correct choices in the first trial was significantly above chance when the correct ramp was blue for *L. delicata* (mean Prob choice = 0.657, p_mcmc_ < 0.05) but not for *L. guichenoti* (mean Prob choice = 0.553, p_mcmc_ = 0.14). When the correct choice was red, it was not significant for neither species (*L. delicata*: mean Prob choice = 0.347, p_mcmc_ = 0.55; *L. guichenoti*: mean Prob choice = 0.347, p_mcmc_ = 0.55).

#### Light spectrum

To test if the bias towards blue was something acquired during training, we compared the light spectrum of the ramps used in the associative task and the white ones used during habituation. We took three measurements of ten ramps per color with a spectophotometer, and then analyzed the spectrum and the perceived differences in color using the package pavo. The spetrum of each type of ramp are shown in [Fig. 4](#fig-spectrum); the perceived chromatic constrasts between ramps are shown in [Fig. 5](#fig-perceived1) and [Fig. 6](#fig-perceived2).

| 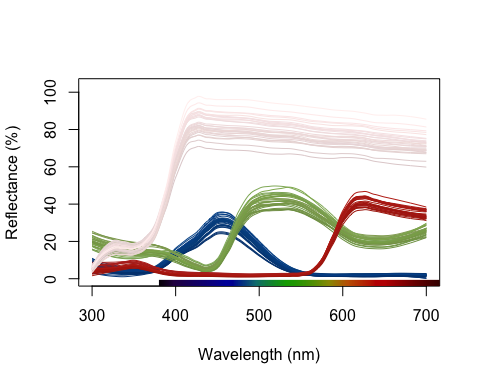  Fig 4— Light spectrum of the ramps used in the associative task and the white ones used during habituation. The different colors represent the different ramps. |
| --- |

| 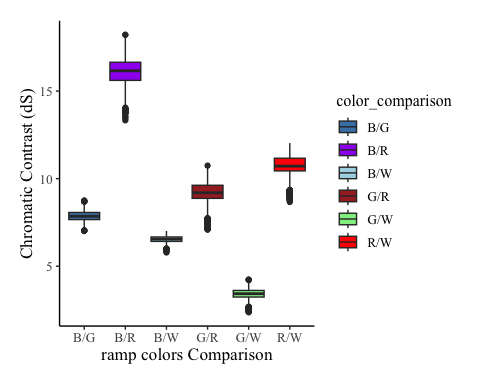  Fig 5— Perceived chromatic contrasts between ramps. |
| --- |

| 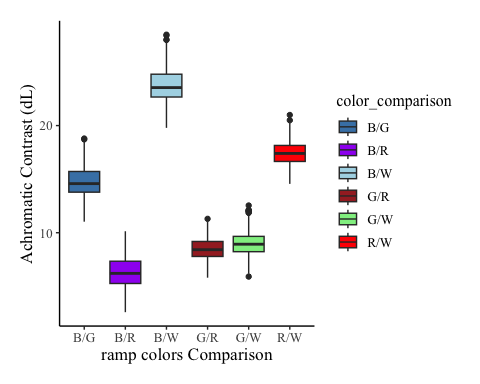  Fig 6— Perceived achromatic contrasts between feeders. |
| --- |

#### Checking the models plots

Model formula for task is:
Choice ~ Trial * cort * temp + (1 + Trial|lizard_id)
Plots for the different models of the associative task:
1.- *L. delicata*
1.a.- Red

Estimate Est.Error Q2.5 Q97.5
R2 0.1695644 0.02196313 0.1255778 0.2110525

| 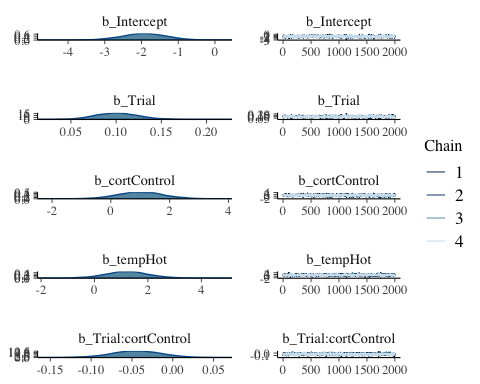 |
| --- |
| 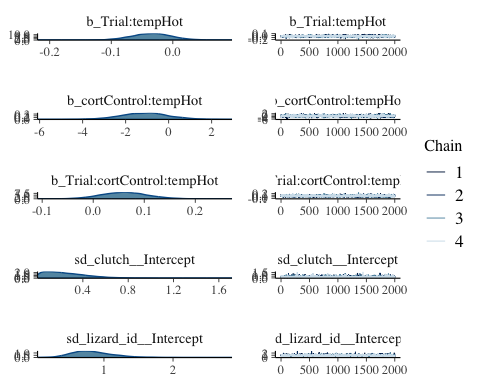 |
| 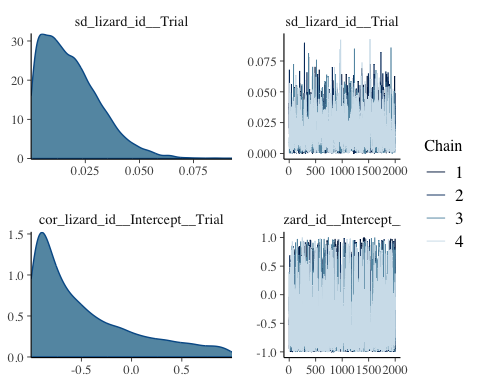 |

1.b.- Blue

Estimate Est.Error Q2.5 Q97.5
R2 0.07757601 0.02088415 0.03926128 0.1200952

| 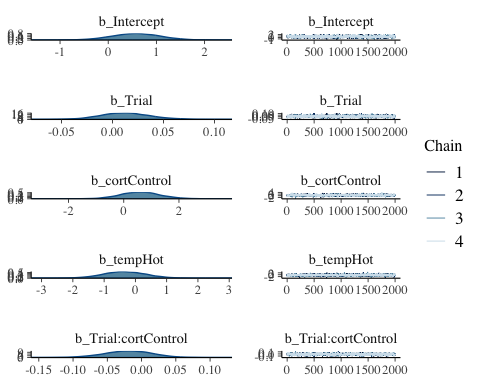 |
| --- |
| 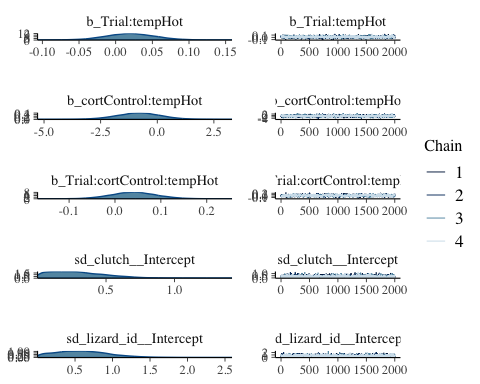 |
| 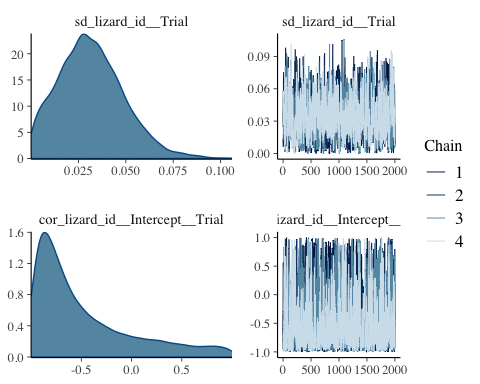 |

2.- *L. guichenoti*
2.a.- Red

Estimate Est.Error Q2.5 Q97.5
R2 0.1858621 0.02466494 0.1359576 0.2330882

| 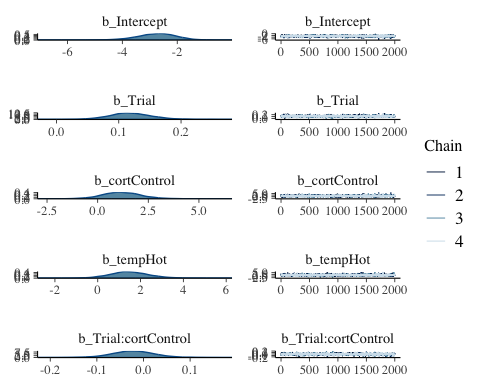 |
| --- |
| 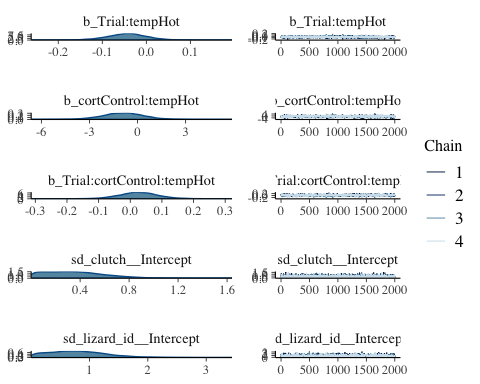 |
| 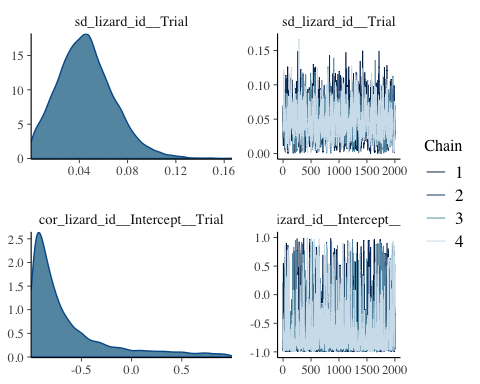 |

2.b.- Blue

Estimate Est.Error Q2.5 Q97.5
R2 0.1367205 0.02551682 0.08796974 0.1865906

| 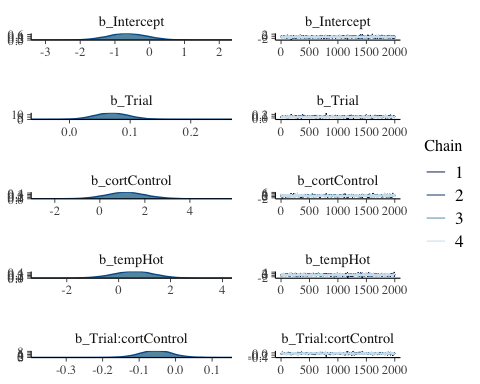 |
| --- |
| 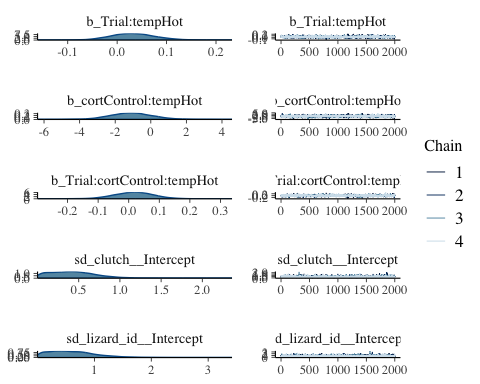 |
| 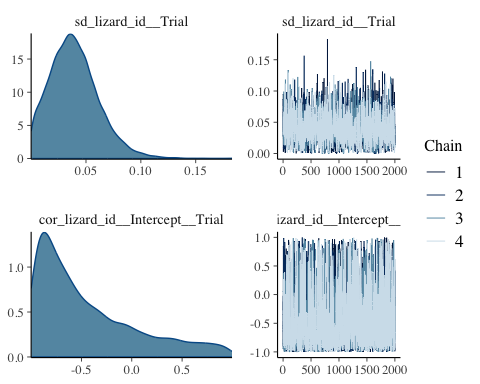 |

#### Models with age included

Table 3. Results for *L. delicata* assigned to red ramps when the model included the age:

| Predictors | Estimate | Est.Error | l-95% CI | u-95% CI | Rhat | Bulk_ESS | Tail_ESS |
| --- | --- | --- | --- | --- | --- | --- | --- |
| Intercept | -2.16 | 0.66 | -3.49 | -0.89 | 1.00 | 3402.24 | 3887.20 |
| age.start | 0.00 | 0.02 | -0.03 | 0.04 | 1.00 | 5418.98 | 5776.45 |
| trial_associative | 0.10 | 0.02 | 0.06 | 0.15 | 1.00 | 3692.82 | 4207.48 |
| cortControl | 1.09 | 0.86 | -0.60 | 2.83 | 1.00 | 3201.98 | 3890.41 |
| tempHot | 1.28 | 0.93 | -0.47 | 3.15 | 1.00 | 2934.34 | 3471.54 |
| trial_associative:cortControl | -0.05 | 0.03 | -0.11 | 0.01 | 1.00 | 3572.26 | 4634.15 |
| trial_associative:tempHot | -0.04 | 0.03 | -0.10 | 0.03 | 1.00 | 3574.66 | 4356.48 |
| cortControl:tempHot | -1.26 | 1.21 | -3.73 | 1.08 | 1.00 | 3074.88 | 4021.52 |
| trial_associative:cortControl:tempHot | 0.06 | 0.04 | -0.03 | 0.15 | 1.00 | 3628.87 | 4183.50 |

Table 4. Results for *L. delicata* assigned to blue ramps when the model included the age:

| Predictors | Estimate | Est.Error | l-95% CI | u-95% CI | Rhat | Bulk_ESS | Tail_ESS |
| --- | --- | --- | --- | --- | --- | --- | --- |
| Intercept | 0.50 | 0.58 | -0.64 | 1.64 | 1.00 | 3044.27 | 4311.87 |
| age.start | -0.00 | 0.01 | -0.01 | 0.01 | 1.00 | 5860.40 | 5710.61 |
| trial_associative | 0.01 | 0.02 | -0.04 | 0.06 | 1.00 | 3068.62 | 4168.88 |
| cortControl | 0.70 | 0.82 | -0.88 | 2.34 | 1.00 | 2870.11 | 3815.82 |
| tempHot | -0.34 | 0.79 | -1.93 | 1.22 | 1.00 | 2598.17 | 3477.53 |
| trial_associative:cortControl | -0.02 | 0.04 | -0.09 | 0.05 | 1.00 | 2915.98 | 3893.50 |
| trial_associative:tempHot | 0.02 | 0.03 | -0.05 | 0.09 | 1.00 | 2593.93 | 3764.25 |
| cortControl:tempHot | -0.98 | 1.13 | -3.23 | 1.25 | 1.00 | 2537.70 | 3924.03 |
| trial_associative:cortControl:tempHot | 0.04 | 0.05 | -0.06 | 0.14 | 1.00 | 2642.40 | 4091.36 |

Table 5. Results for *L. guichenoti* assigned to red ramps when the model included the age:

| Predictors | Estimate | Est.Error | l-95% CI | u-95% CI | Rhat | Bulk_ESS | Tail_ESS |
| --- | --- | --- | --- | --- | --- | --- | --- |
| Intercept | -3.06 | 0.84 | -4.87 | -1.55 | 1.00 | 2641.91 | 2963.70 |
| age.start | -0.00 | 0.02 | -0.04 | 0.03 | 1.00 | 5914.10 | 5309.44 |
| trial_associative | 0.12 | 0.04 | 0.06 | 0.20 | 1.00 | 2411.12 | 2897.00 |
| cortControl | 1.28 | 1.15 | -0.97 | 3.62 | 1.00 | 2479.35 | 3043.41 |
| tempHot | 1.65 | 1.14 | -0.57 | 3.97 | 1.00 | 2336.65 | 2802.60 |
| trial_associative:cortControl | -0.02 | 0.05 | -0.13 | 0.08 | 1.00 | 2444.48 | 3222.97 |
| trial_associative:tempHot | -0.05 | 0.05 | -0.15 | 0.04 | 1.00 | 2253.20 | 2466.35 |
| cortControl:tempHot | -1.03 | 1.53 | -4.17 | 1.91 | 1.00 | 2353.37 | 3274.21 |
| trial_associative:cortControl:tempHot | 0.03 | 0.07 | -0.11 | 0.17 | 1.00 | 2319.80 | 3341.04 |

Table 6. Results for *L. guichenoti* assigned to blue ramps when the model included the age:

| Predictors | Estimate | Est.Error | l-95% CI | u-95% CI | Rhat | Bulk_ESS | Tail_ESS |
| --- | --- | --- | --- | --- | --- | --- | --- |
| Intercept | -0.61 | 0.73 | -2.07 | 0.85 | 1.00 | 4476.16 | 4928.86 |
| age.start | 0.01 | 0.01 | -0.02 | 0.03 | 1.00 | 5957.56 | 5181.70 |
| trial_associative | 0.07 | 0.03 | 0.01 | 0.13 | 1.00 | 4004.25 | 4029.03 |
| cortControl | 1.32 | 1.00 | -0.70 | 3.29 | 1.00 | 3729.95 | 4199.07 |
| tempHot | 0.38 | 0.94 | -1.61 | 2.22 | 1.00 | 4055.41 | 4399.38 |
| trial_associative:cortControl | -0.05 | 0.05 | -0.14 | 0.04 | 1.00 | 3437.88 | 3686.90 |
| trial_associative:tempHot | 0.03 | 0.04 | -0.05 | 0.12 | 1.00 | 3667.39 | 4011.10 |
| cortControl:tempHot | -1.23 | 1.34 | -3.86 | 1.52 | 1.00 | 3524.34 | 4053.85 |
| trial_associative:cortControl:tempHot | 0.02 | 0.06 | -0.12 | 0.14 | 1.00 | 3340.33 | 4029.47 |

| 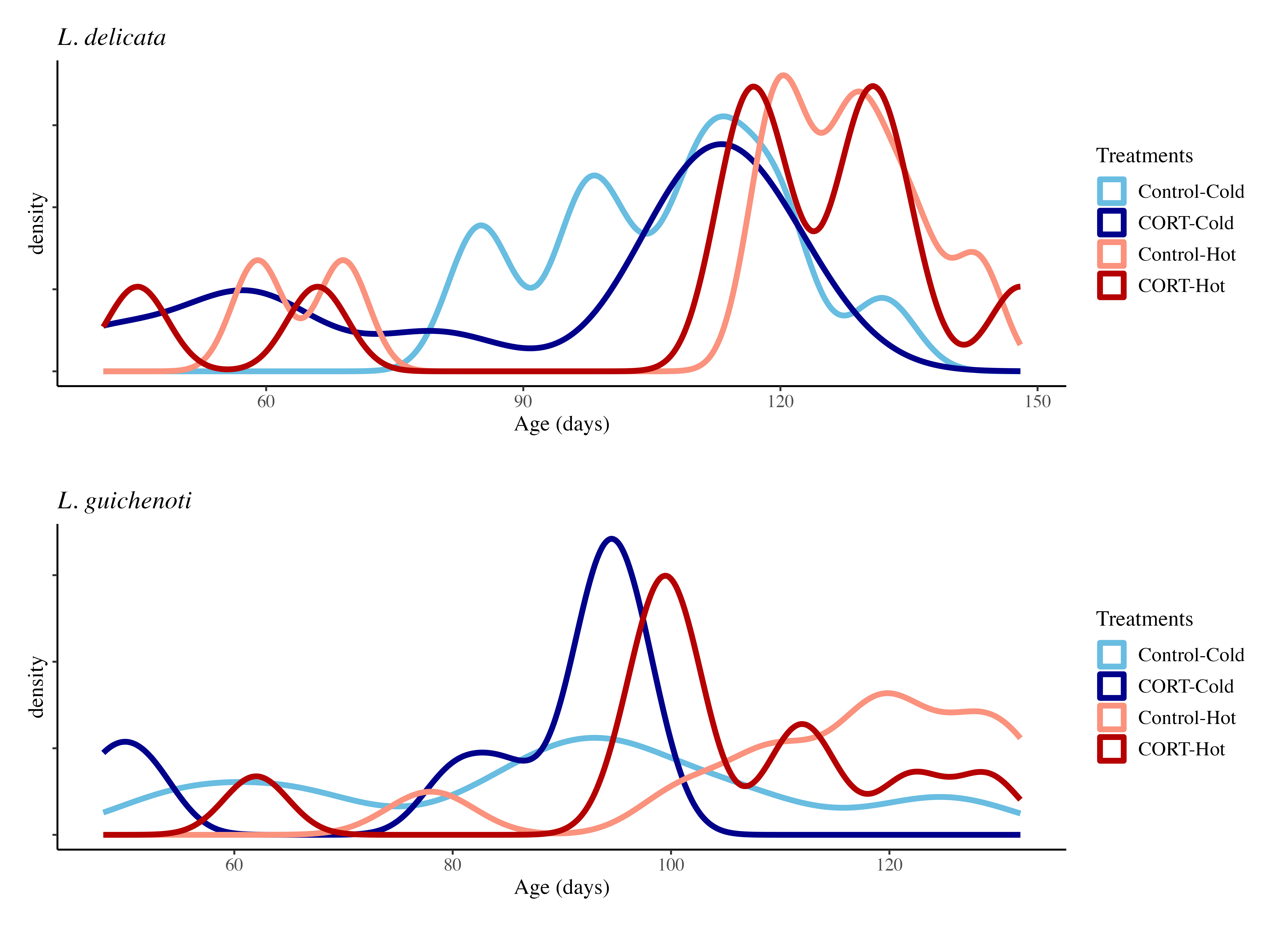  Fig 7— Distribution of the age of the lizards by treatment and species |
| --- |
